# Granulomatous Inflammation and Hypercalcemia in Patients With Severe Systemic Oxalosis

**DOI:** 10.1016/j.ekir.2021.11.020

**Published:** 2021-11-24

**Authors:** Peggy Perrin, Jérome Olagne, Arnaud Delbello, Stanislas Bataille, Laurent Mesnard, Claire Borni, Bruno Moulin, Sophie Caillard

**Affiliations:** 1 Department of Nephrology and Transplantation, University Hospital, Strasbourg, France; 2 Fédération de Médecine Translationnelle (FMTS), Strasbourg, France; 3 Institut National de la Santé et de la Recherche Médicale (INSERM) U1109, LabEx TRANSPLANTEX, Strasbourg, France; 4 Department of Pathology, University Hospital, Strasbourg, France; 5 Département de Néphrologie, Dialyse et Transplantation d’Organes, Centre Hospitalier et Universitaire de Toulouse, Toulouse, France; 6 Institut National de la Santé et de la Recherche Médicale—Centre de Physiopathologie Toulouse Purpan, Institut National de la Santé et de la Recherche Médicale (INSERM) UMR 1043-Centre National de le Recherche Scientifique (CNRS) 5282, Toulouse, France; 7 Université Paul Sabatier Toulouse III, Toulouse, France; 8 Phocean Institute of Nephrology, Marseille, France; 9 ELSAN, Clinique Bouchard, Marseille, France; 10 Aix-Marseille Univ, C2VN, Institut National de la Santé et de la Recherche Médicale (INSERM), Institut National de la Recherche Agronomique (INRAE), Marseille, France; 11 Service des Soins Intensifs Néphrologiques et Rein Aigu, Department of Nephrology and Transplantation, Hôpital Tenon, Assistance Publique–Hôpitaux de Paris (APHP) Sorbonne Université, Paris, France; 12 AURAL, Colmar, France

## Introduction

Primary hyperoxalurias (PHs) are rare genetic disorders that cause hyperproduction of oxalate in the liver. The clinical manifestations of excess oxalate include urolithiasis, nephrocalcinosis, and end-stage renal disease. The onset of renal failure—which typically represents a turning point in the course of PHs—results in the inability to remove oxalate in the urine. This in turn leads to a progressive accumulation of insoluble calcium-oxalate (Ca-Ox) crystals in various tissues. This severe condition, termed systemic oxalosis (SO),^1^ has an unfavorable prognosis characterized by persistent pain, increased vulnerability to fractures,^2^ and significant mortality. SO is currently thought to result mainly from diagnostic delays or limited access to optimal care. A clinically feasible way to reduce the risk of SO in patients with PHs is simultaneous liver–kidney transplantation (SLKT) performed at an early disease stage. A novel therapeutic approach lies in the use of RNA interference therapy—which may be especially well suited at reducing the hepatic hyperproduction of oxalate.^3^

Data from animal models have suggested that inflammation may play a role in the pathogenesis of oxalosis.^4^ In addition, bone accumulation of Ca-Ox crystals in patients with SO has been found to elicit the formation of granulomas^2^—whose presence may be related to the onset of hypercalcemia.^5^ Notwithstanding these preliminary observations, the clinical impact of inflammation and hypercalcemia during the course of SO has not been fully elucidated. This multicenter case series was aimed at investigating this issue in 5 adult patients with PH complicated by end-stage renal disease. ^18^F-fluorodeoxyglucose positron-emission tomography/computed tomography (FDG-PET/CT) data, and histology, and laboratory findings were obtained in the context of clinical care and retrospectively reviewed and analyzed in relation to clinical outcomes.

## Case Presentation

The 5 study patients were 3 men and 2 women recruited from 4 French Nephrology centers. The median age at the beginning of dialysis was 34 years (range: 25–62 years). The diagnosis of PH type 1 and PH type 2 was confirmed by mutation analysis of the *AGXT* gene in patients number (#)1, #2, #4, and #5 and of the *GRHP* gene in patient #3, respectively. Of the 5 study patients, 3 were diagnosed with having PH after the initiation of dialysis. The median duration of dialysis before LT/SLKT was 7 years (range: 4–9 years). Patient follow-up was concluded in November 2020. The median follow-up time after transplantation was 24 months (range: 16–54 months). The general characteristics, laboratory findings, and clinical outcomes of the study patients are summarized in Table 1.

**Table 1:** General characteristics, clinical outcomes, laboratory findings, and treatment approaches for hypercalcemia in the 5 study patients

	Patient #1	Patient #2	Patient #3	Patient #4	Patient #5
Sex	F	M	M	M	F
PH type	1	1	2	1	1
Age at diagnosis, yr (circumstances of diagnosis)	67 (spinal cord compression by Ca-Ox deposits)	5 (nephrocalcinosis)	Teenager (urolithiasis)	35 (kidney graft failure)	51 (pre-KT evaluation)
Renal presentation	Urolithiasis, ESRD	Nephrocalcinosis, urolithiasis, ESRD	Urolithiasis, ESRD	ESRD	Urolithiasis, ESRD
Age at the beginning of dialysis, yr (calendar year)	62, 2009	25, 2010	34, 2009	33, 2013	47, 2012
Dialysis duration before SLKT/LT, yr	7	8	9	4	7
Transplantation, type and date	SLKT in 2016	SLKT in 2018	KT in 2016, SLKT in 2018	KT in 2015, LT in 2017	SLKT in 2019
Clinical outcomes					
Kidney graft outcome					
Early outcome	DGF	Primary dysfunction	Early failure of the first kidney graft due to recurrent crystal nephropathy	Early graft failure due to graft venous thrombosis	DGF
Graft crystals on biopsy	Yes	Yes	Yes	Yes	Yes
Graft lithiasis	Yes (obstructive lithiasis requiring long-term pyelostomia)	No	No	No	No
GFR (ml/min per 1.73 m^2^) at last follow-up	15	Hemodialysis	28	Hemodialysis	23
Bone outcomes					
Fractures (localization)	Yes (foot)	Yes (vertebra)	Yes (vertebra, humerus)	Yes (vertebra)	No
Rheumatologic pain	Pain (spine, extremities) that led to an impaired mobility; appearance 3 years after the beginning of dialysis Spinal cord compression	Intense pain that limited mobility (wheelchair) and that required treatment with morphine; appearance 5 years after the beginning of dialysis	Rheumatic pain	Chronic pain, knee crystalline arthritis, Achilles tendon rupture	No
Cardiovascular events	Calcific aortic stenosis requiring valve replacement, thrombosis of the fistula	Transient ischemic attack, calcific mitral stenosis requiring valve replacement, thrombosis of the fistula	Calcific mitralic stenosis with heart disease, thrombosis of the fistula	Thrombosis of the fistula, lower limb DVT, pulmonary embolism	Thrombosis of the fistula
Other clinical manifestations	—	Cutaneous necrosis, tophi on fingers, hepatosplenomegalia	Cutaneous necrosis	Pancytopenia	Cutaneous necrosis
Death (date, cause)	No	Yes (2020, calcific mitral stenosis, sepsis, cachexia, severe hypercalcemia)	Yes (2020, sepsis)	Yes (2020, sepsis, angiocholitis, cachexia)	No
Laboratory findings					
Hypercalcemia,^a^ years of onset after the beginning of dialysis	Yes, 3 (2012)	Yes, 4 (2014)	Yes, 5 (2014)	Yes, 4 (2017)	Yes, NA
Hypoalbuminemia (<30 g/l)	Yes	Yes	Yes	Yes	Yes
Chronic elevation of C-reactive protein	No	Yes	Yes	Yes	No
Angiotensin-converting enzyme	Elevated	Elevated	Upper limit of normal	Elevated	Elevated
1,25 OH_2_-vitaminD	Normal or transiently elevated	Elevated	Elevated	Elevated for a patient on dialysis	Low
Bone remodeling markers	Elevated	Elevated	Elevated	Elevated	Elevated
Treatment for hypercalcemia	Bisphosphonates, denosumab 60 mg every 2–3 mo	Denosumab 60 mg and pamidronate	Bisphosphonates every 2 mo followed by denosumab 60 mg every 3 mo	Pamidronate in 2020	No
Treatment for suspected hyperparathyroidism	Cinacalcet 30 mg/d after LKT	No	Parathyroidectomy in 2015 leading to hypoparathyroidism; no correction of hypercalcemia	Cinacalcet before LT	No

#, number; Ca-Ox, calcium-oxalate; DGF, delayed graft function; DVT, deep vein thrombosis; ESRD, end-stage renal disease; F, female; KT, kidney transplantation; LT, liver transplantation; LKT, liver–kidney transplantation; M, male; NA, not available; PH, primary hyperoxaluria; SLKT, simultaneous liver–kidney transplantation.

a Hypercalcemia was defined by a serum ionized calcium > 1.3 mmol/l or a serum corrected calcium > 2.6 mmol/l (>10.4 mg/dl).

### Imaging Findings

**Figure 1:**
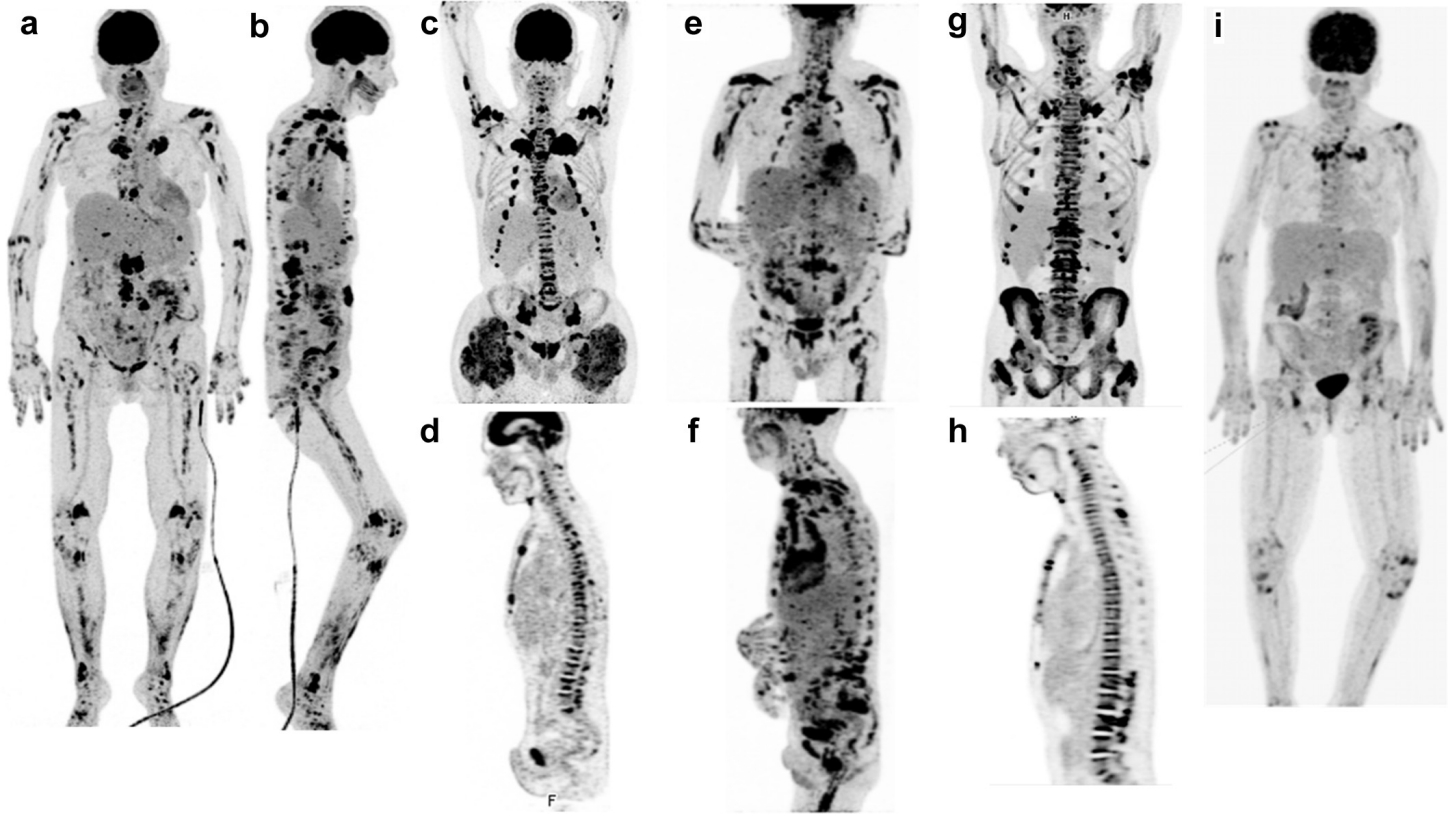
Illustrative FDG positron-emission tomography/computed tomography images in the 5 study patients. The FDG positron-emission tomography/computed tomography whole-body images for patients (a, b) #1, (c, d) #2, (e, f) #3, (g, h) #4, and (i) #5 revealed bilateral diffuse hypermetabolic lesions throughout the entire skeleton. In addition, numerous joints were affected (shoulder girdle joints, chondrocostal and costovertebral joints, spine joints, pelvic girdle joints, and limb joints). Patients (c, d) #2, (e, f) #3, and (g, h) #4 had evidence of spondylitis; we also identified the presence of sacroiliitis in patients (c) #2, (e) #3, and (g) #4. Foci of increased FDG uptake were identified at multiple muscle insertions, cutaneous areas, and cartilages (larynx). (c) Patient #2 had bulk hypermetabolic calcified muscular masses at the hip. (i) The intensity and number of hypermetabolic lesions were lower for patient #5. #, number; FDG, ^18^F-fluorodeoxyglucose.

The initial FDG-PET/CT scans were performed 4 and 10 months before LT/SLKT and 1, 12, and 15 months after transplantation in patients #2, #4, #5, #1, and #3, respectively (Figure 1 and Supplementary Figure S1). All participants were found to have diffuse joint, bone, and soft tissue hypermetabolic lesions in areas of calcium depositions. Osseous lesions of the spine either involved the vertebral body (with evidence of spondylitis in patients #2, #3, and #4) or the posterior elements (leading to spinal cord compression in patient #1). A biopsy of a hypermetabolic vertebral lesion in patient #1 revealed the presence of an inflammatory granulomatous reaction elicited by Ca-Ox crystals (Supplementary Figure S2, panels K–O). CT images obtained using the “bone window” settings identified the presence of reactive bone resorption areas adjacent to the Ca-Ox deposits, which were associated with fractures. A longitudinal scan performed in patient #2 revealed a rapid progression of valvular and vascular calcifications (Supplementary Figure S1). Although repeated FDG-PET/CT examinations after SLKT revealed that hypermetabolic lesions tended to persist over time (as long as 4 post-transplant years in patient #1), FDG avidity of the foci decreased significantly. Bone scintigraphy in patients #1 and #4 revealed mild tracer uptake in bone areas surrounding calcium deposition, which corresponded to the bone-resorptive lesions (Supplementary Table S1).

### Laboratory Findings

Hypoalbuminemia and hypercalcemia were observed in all participants (Table 1 and Supplementary Table S2). Although the presence of hypercalcemia was reflected by high serum ionized calcium or corrected calcium levels, total serum calcium was generally within the reference range. Patients #1 and #2 developed hypercalcemia after 3 and 4 years on dialysis, respectively; the event was concomitant to the onset of bone pain and persisted at follow-up. A complete diagnostic workup ruled out the presence of malignancies. Because of hypercalcemia and end-stage renal disease, hyperparathyroidism was frequently suspected. Nevertheless, specific drug treatments were ineffective at lowering hypercalcemia—suggesting it was unrelated to parathyroid hormone. Increased ionized calcium was accompanied by elevated serum biomarkers of bone turnover.

In addition, biomarkers of chronic granulomatous disease—including serum levels of angiotensin converting enzyme and/or 1,25OH_2_D—were elevated in all participants. Patients #2, #3, and #4 were found to have laboratory signs of a chronic inflammatory syndrome. Serum levels of interleukin-6, interleukin-1, and tumor necrosis factor alfa were quantified in 3 cases and interleukin-6 was consistently increased. Elevated serum oxalate levels did not generally regress in the post-transplantation period and—in patient #1—they were still evident at 4 years after SKLT. Patients #1 and #2 were found to have increased urinary oxalate and calcium concentrations coupled with crystalluria (whewellite and/or weddellite).

### Histology Results

Within the context of clinical care, patients #1, #2, #4, and #5 underwent biopsies of hypermetabolic lesions located in different tissues (bone and soft tissues; Supplementary Table S1). Histology examination revealed the presence of reactive granulomas consisting of both multinucleate giant cells and macrophages that typically surrounded Ca-Ox crystals (Supplementary Figure S3). Multinucleate giant cell in contact with the trabecular surfaces exhibited an osteoclast-like activity, which led to bone resorption. Immunohistochemistry results of bone and node biopsies (patient #1) and of hypermetabolic calcified muscle lesions (patient #2) revealed that multinucleate giant cell abundantly expressed CD68 and RANK-L (Supplementary Figure S2).

### Clinical Course (Table 1)

All participants had recurrence of crystal nephropathy in the kidney graft. Patient #1 had obstructive calculi on the kidney graft. Recurrence of nephropathy leads to early renal failure after the first kidney transplantation in patients #4 and #3 (who had a diagnosis of type 2 PH).^6^ At the end of the follow-up, 2 patients lost their kidney graft and the remaining 3 had severe renal dysfunction. Diffuse valvular calcifications—which were likely related to hypercalcemia—required replacement valve surgery in patients #1 and #2. All cases had a history of venous thrombosis, and cutaneous necrosis occurred in 3 patients. Furthermore, 4 patients had fractures and rheumatic pain. Pain slowly regressed after transplantation in parallel with the reduction of FDG avidity in hypermetabolic foci on FDG-PET/CT. The clinical outcomes were generally unfavorable. Patients #2, #3, and #4 died of sepsis and/or cachexia at the age of 35, 43, and 40 years, respectively.

#### Treatment Approach of Hypercalcemia, Fractures, and Crystal Rheumatism

Although patients #1, #2, #3, and #4 received bone-antiresorptive agents (bisphosphonates and/or denosumab) for hypercalcemia and fractures after SLKT, their efficacy in normalizing calcium levels was transient (Table 1). Patients #1, #2, and #3 were treated with subcutaneous injections of denosumab—a monoclonal antibody against RANK-L; this approach effectively but temporarily reduced serum calcium and bone turnover biomarkers. Consequently, a prolonged treatment was necessary. Patients #1, #2, and #3 received high-dose corticosteroids during LT/SLKT induction and/or because of acute graft rejection. Although steroids induced a temporary remission of hypercalcemia, relapses were evident when these drugs were tapered off.

Patient #2 received solumedrol boluses at induction—which led to a dramatic reduction of crystal arthropathy-associated pain and inflammation. Unfortunately, the disabling pain relapsed after a few months. A further therapeutic attempt with infliximab was unsuccessful, whereas the interleukin-1 receptor antagonist anakinra was rapidly withdrawn owing to infectious complications of cutaneous tophi.

## Discussion

Please refer to the Supplementary Discussion for further details.

In conclusion, our data illustrate for the first time the key role played by reactive granulomatous inflammation as a key driver for the high morbidity (i.e., crystal arthropathy, fractures, recurrent crystal nephropathy, and cardiovascular complications) and mortality observed in patients with severe SO. FDG-PET/CT and biological markers may help clinicians to evaluate the severity of oxalosis and the risk of recurrent crystal nephropathy after kidney transplantation (Table 2).

**Table 2:** Teaching points

• PH—a rare genetic disease characterized by the hepatic hyperproduction of oxalate that frequently leads to ESRD.
• After renal failure, insoluble calcium oxalate crystals accumulate in various tissues—mainly in the bone—resulting in systemic oxalosis. Systemic oxalosis has unfavorable outcomes characterized by persistent pain, fractures, and significant mortality.
• Early management of patients with PH and ESRD is paramount to avoid severe systemic oxalosis and recurrence of crystal nephropathy in the kidney allograft.
• Although current strategies to treat patients with PH with ESRD mainly consist of dual liver–kidney transplantation, RNA interference therapy will likely lead to more tailored treatment options.
• The role of inflammation in the pathogenesis of systemic oxalosis and in its clinical manifestations remains unclear.
• In this case series of 5 patients with severe oxalosis, FDG-PET/CT images revealed diffuse joint, bone, and soft tissue hypermetabolic lesions, which corresponded to granulomas elicited by calcium-oxalate crystals. Hypermetabolic lesions—which were associated with bone resorption and fractures—did not regress after kidney transplantation. Laboratory findings revealed hypoalbuminemia and hypercalcemia accompanied by increased bone turnover and granulomatosis biomarkers. The clinical course was unfavorable, and 3 patients died of the disease. Collectively, these data illustrate the key role played by granulomatous inflammation as a driver of the high morbidity and mortality in patients with severe systemic oxalosis.
• Although bone antiresorptive agents and corticosteroids were effective in controlling hypercalcemia, they did not prevent recurrences.
• FDG-PET/CT may serve as a promising imaging tool to evaluate the systemic burden of oxalosis.

ESRD, end-stage renal disease; FDG-PET/CT,^18^F-fluorodeoxyglucose positron-emission tomography/computed tomography; PH, primary hyperoxaluria.

## Disclosure

BM serves as an advisory board member and received research grants from Alnylam Pharmaceuticals. The remaining authors declared no competing interests.

## Patient Consent

This is a retrospective review of prospectively collected data in the context of clinical care. According to current French regulations, the need for institutional review board approval was waived owing to the study design. Written informed consent for additional analyses of biopsies (immunohistochemistry) for research purposes was obtained from patients #1 and #2; these analyses were approved by the local institutional review board (approval number: DC-2013-1990).

## Supplementary Material

Supplementary File (PDF)

**Figure S1.** Illustrative ^18^F-fluorodeoxyglucose positron-emission tomography/computed tomography (FDG-PET/CT) images in patients #2 (a, b, g, h, i, j, m, n) and #3 (c, d, e, f). Patterns of FDG uptake in patient #2: spinal images revealed the presence of spondylitis with a maximum standardized uptake value (SUVmax) of 14.6. Areas of increased tracer uptake were also noted in the sacroileal (SUVmax: 16.7), gluteal muscles (SUVmax: 7.5; a), sternoclavicular joints (g), pelvis girdle (i), and hip joint (h). Axial image revealing hypermetabolic lesions in native kidneys (m). Patient #2 presented hepatosplenomegaly with slightly increased FDG uptake (a). The corresponding CT images (“bone window” protocol) revealed that the hypermetabolic foci on PET were extensive calcium deposits (b, j, d, f); bone resorption was evident in the adjacent bone (e.g., endplate of the vertebrae [k] or cortical bones of the limbs [f]). Patient #3 had humeral hypermetabolic lesions (c, e) accompanied by bone erosion and thinning of the adjacent cortical area (d, f); these lesions resulted in a fracture of the right humerus (→; c, d). Patient #3 had severe aortic calcifications (k), whereas severe mitral valve calcifications were found in patient #1 (n).

**Figure S2.** Immunohistochemical analysis of tissue inflammatory cells using the following markers: CD68 (column 2), CD163 (column 3), RANK (column 4), and RANK-L (column 5). Panels A–E: Patient #2. On analyzing biopsies obtained from hypermetabolic calcified muscular masses, multinucleated giant cells surrounding Ca-Ox deposits were evident (A; hematoxylin and eosin staining, 400× magnification). Giant cells adjacent to calcifications were positive for CD68 (B; 200× magnification). Although we found a weak RANK expression (D; 400× magnification), RANK-L was abundantly expressed (E; 400× magnification). CD163-positive cells were identified in the proximity of granulomatous lesions (C; 200× magnification). F–J: Patient #1 had a bone exostosis in the right hallux front. An osteoid matrix without osteoblasts but accompanied by an inflammatory infiltrate was identified adjacent to calcium-oxalate crystals (F; hematoxylin and eosin staining, 200× magnification). Immunochemical staining of multinucleated giant cells revealed that marker expression levels were as follows: high CD68 (G; 400× magnification), low CD163 (H; 400× magnification), low RANK (I; 400× magnification), and high RANK-L (J; 400× magnification). K–O: L3–L4 vertebral bone biopsy obtained from patient #1 revealing the presence of numerous Ca-Ox crystals surrounded by inflammatory cells (F; hematoxylin and eosin staining, 200× magnification). Immunochemical staining revealed that marker expression levels were as follows: high CD68 (K; 400× magnification), low CD163 (H; 400× magnification), low RANK (I; 400× magnification), and high RANK-L (J; 400× magnification). P–T: Iliac bone biopsy obtained from patient #1 revealing the presence of Ca-Ox crystals in bone morrow accompanied by a granulomatous reaction (K; hematoxylin and eosin staining, 400× magnification). Immunochemical staining of multinucleated giant cells that surrounded the Ca-Ox crystals revealed that marker expression levels were as follows: high CD68 (L; 400× magnification), high RANK-L (O; 400× magnification), low CD163 (R; 400× magnification), and low RANK (N; 400× magnification). U–Y: Lymph node biopsy obtained from patient #1: very large Ca-Ox crystal (U; hematoxylin and eosin staining, 200× magnification). Immunochemical staining of multinucleated giant cells revealed that marker expression levels were as follows: high CD68 (V; 400× magnification), low CD163 (W; 400× magnification), low RANK (X; 400× magnification), and high RANK-L (Y; 400× magnification). The primary antibodies used for the detection of RANK and RANKL were obtained from LSBio (Seattle, WA, USA; catalog numbers: LS-B11252 and LS-B1425, respectively).

**Figure S3.** Illustrative biopsy findings in 3 study patients. Panel A. Patient #4: Iliac bone biopsy revealing the presence of multinucleated giant cells (MGCs) in proximity to Ca-Ox crystals (arranged in a star-like figure) adjacent to spongy bone trabeculae. When in contact to the trabecular surface, MGC acted in an osteoclast-like fashion and led to an increased percentage of the eroded surface. Osteoid tissue was observed between the crystals and the bone (hematoxylin-eosin-saffron staining; 400× magnification). Panel B. Patient #1: Iliac bone biopsy revealing the presence of Ca-Ox crystal calcifications surrounded by a reactive inflammatory infiltrate that eroded the trabecular bone (→) (hematoxylin and eosin staining, 400× magnification). Panel C. Patient #2: Gluteal mass biopsy revealing the presence of diffused calcifications surrounded by a reactive inflammatory infiltrate with numerous MGC (hematoxylin and eosin staining, 400× magnification).

**Table S1.** Summary of imaging (FDG-PET/CT and bone scintigraphy) and histology findings in 5 patients with severe oxalosis.

**Table S2.** Laboratory findings in the 5 study patients.

**Supplement Discussion**

**STROBE Statement** (PDF)
