# Supplementary material for: Granulomatous Inflammation and Hypercalcemia in Patients With Severe Systemic Oxalosis
Source: Kidney Int Rep. 2021 Nov 24;7(2):343–9. doi: 10.1016/j.ekir.2021.11.020 (PMC8820998; doi:10.1016/j.ekir.2021.11.020)
Supplement: Supplementary File (PDF) [file mmc1.pdf]

Supplementary material

Figure legends

Figure S1. Illustrative ^{18}F-fluorodeoxyglucose positron-emission tomography/computed tomography (FDG-PET/CT) images in patients #2 (a, b, g, h, i, j, m, n) and #3 (c, d, e, f)
Patterns of FDG uptake in patient #2: spinal images revealed the presence of spondylitis with a maximum standardized uptake value (SUVmax) of 14.6. Areas of increased tracer uptake were also noted in the sacroileal (SUVmax: 16.7), gluteal muscles (SUVmax: 7.5; a), sterno-clavicular joints (g), pelvis girdle (i), and hip-joint (h). Axial image showing hypermetabolic lesions in native kidneys (m). Patient #2 presented hepatosplenomegaly with slightly increased FDG uptake (a).
The corresponding CT images ("bone window" protocol) revealed that the hypermetabolic foci on PET were extensive calcium deposits (b, j, d, f); bone resorption was evident in the adjacent bone (e.g., endplate of the vertebrae [k] or cortical bones of the limbs [f]).
Patient #3 showed humeral hypermetabolic lesions (c, e) accompanied by bone erosion and thinning of the adjacent cortical area (d, f); these lesions resulted in a fracture of the right humerus (→; c, d).
Patient #3 showed severe aortic calcifications (k), whereas severe mitral valve calcifications were found in patient #1 (n).

Figure S2. Illustrative biopsy findings in three study patients
Panel A. Patient #4: iliac bone biopsy showing the presence of multinucleated giant cells (MGC) in proximity to Ca-Ox crystals (arranged in a star-like figure) adjacent to spongy bone trabeculae. When in contact to the trabecular surface, MGC acted in an osteoclast-like fashion and led to an increased percentage of the eroded surface. Osteoid tissue was observed between the crystals and the bone (hematoxilin-eosin-saffron staining; 400× magnification).
Panel B. Patient #1: iliac bone biopsy showing the presence of Ca-Ox crystals calcifications surrounded by a reactive inflammatory infiltrate that eroded the trabecular bone (→) (hematoxilin and eosin staining, 400× magnification).
Panel C. Patient #2: gluteal mass biopsy showing the presence of diffuse calcifications surrounded by a reactive inflammatory infiltrate with numerous MGC (hematoxilin and eosin staining, 400× magnification).

Figure S3: Immunohistochemical analysis of tissue inflammatory cells using the following markers: CD68 (column 2), CD163 (column 3), RANK (column 4), and RANK-L (column 5)
Panels A-E: Patient #2. On analyzing biopsies obtained from hypermetabolic calcified muscular masses, multinucleated giant cells surrounding Ca-Ox deposits were evident (A; hematoxilin and eosin staining, 400× magnification). Giant cells adjacent to calcifications were positive for CD68 (B; 200× magnification). While we found a weak RANK expression (D; 400× magnification), RANK-L was abundantly expressed (E; 400× magnification). CD163-positive cells were identified in the proximity of granulomatous lesions (C; 200× magnification).
F-J: Patient #1 showed a bone exostosis in the right hallux front. An osteoid matrix without osteoblasts but accompanied by an inflammatory infiltrate was identified adjacent to calcium-oxalate crystals (F; hematoxilin and eosin staining, 200× magnification). Immunochemical staining

of multinucleated giant cells revealed that marker expression levels were as follows: high CD68 (G; 400× magnification), low CD163 (H; 400× magnification), low RANK (I; 400× magnification), and high RANK-L (J; 400× magnification).
K-O: L3−L4 vertebral bone biopsy obtained from patient #1 showing the presence of numerous Ca-Ox crystals surrounded by inflammatory cells (F; hematoxilin and eosin staining, 200× magnification). Immunochemical staining revealed that marker expression levels were as follows: high CD68 (K; 400× magnification), low CD163 (H; 400× magnification), low RANK (I; 400× magnification) and high RANK-L (J; 400× magnification).
P-T: Iliac bone biopsy obtained from patient #1 showing the presence of Ca-Ox crystals in bone morrow accompanied by a granulomatous reaction (K; hematoxilin and eosin staining, 400× magnification). Immunochemical staining of multinucleated giant cells that surrounded the Ca-Ox crystals revealed that marker expression levels were as follows: high CD68 (L; 400× magnification), high RANK-L (O; 400× magnification), low CD163 (R; 400× magnification), low RANK (N; 400× magnification).
U-Y: Lymph node biopsy obtained from patient #1 1: very large Ca-Ox crystal (U; hematoxilin and eosin staining, 200× magnification). Immunochemical staining of multinucleated giant cells revealed that marker expression levels were as follows: high CD68 (V; 400× magnification), low CD163 (W; 400× magnification), low RANK (X; 400× magnification) and high RANK-L (Y; 400× magnification).
The primary antibodies used for the detection of RANK and RANKL were obtained from LSBio (Seattle, WA, USA; catalogue number: LS-B11252 and LS-B1425, respectively).

Figure S1

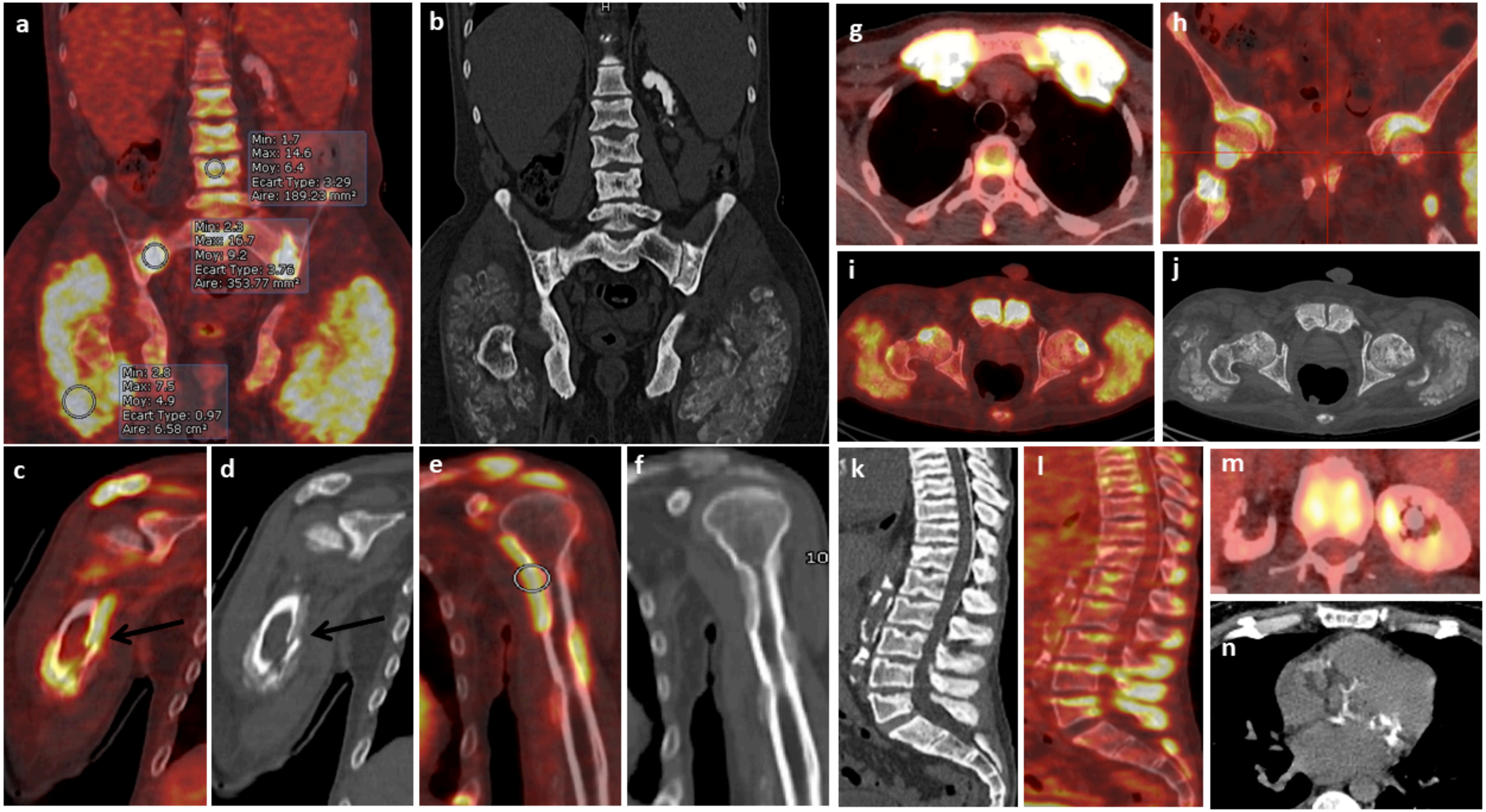
a
Min: 1.7
Max: 14.6
Moy: 6.4
Ecart Type: 3.29
Aire: 189.23 mm²
Min: 2.3
Max: 16.7
Moy: 9.2
Ecart Type: 3.76
Aire: 353.77 mm²
Min: 2.8
Max: 7.5
Moy: 4.9
Ecart Type: 0.97
Aire: 6.58 cm²
b
c
d
e
f
g
h
i
j
k
l
m
n

Figure S2

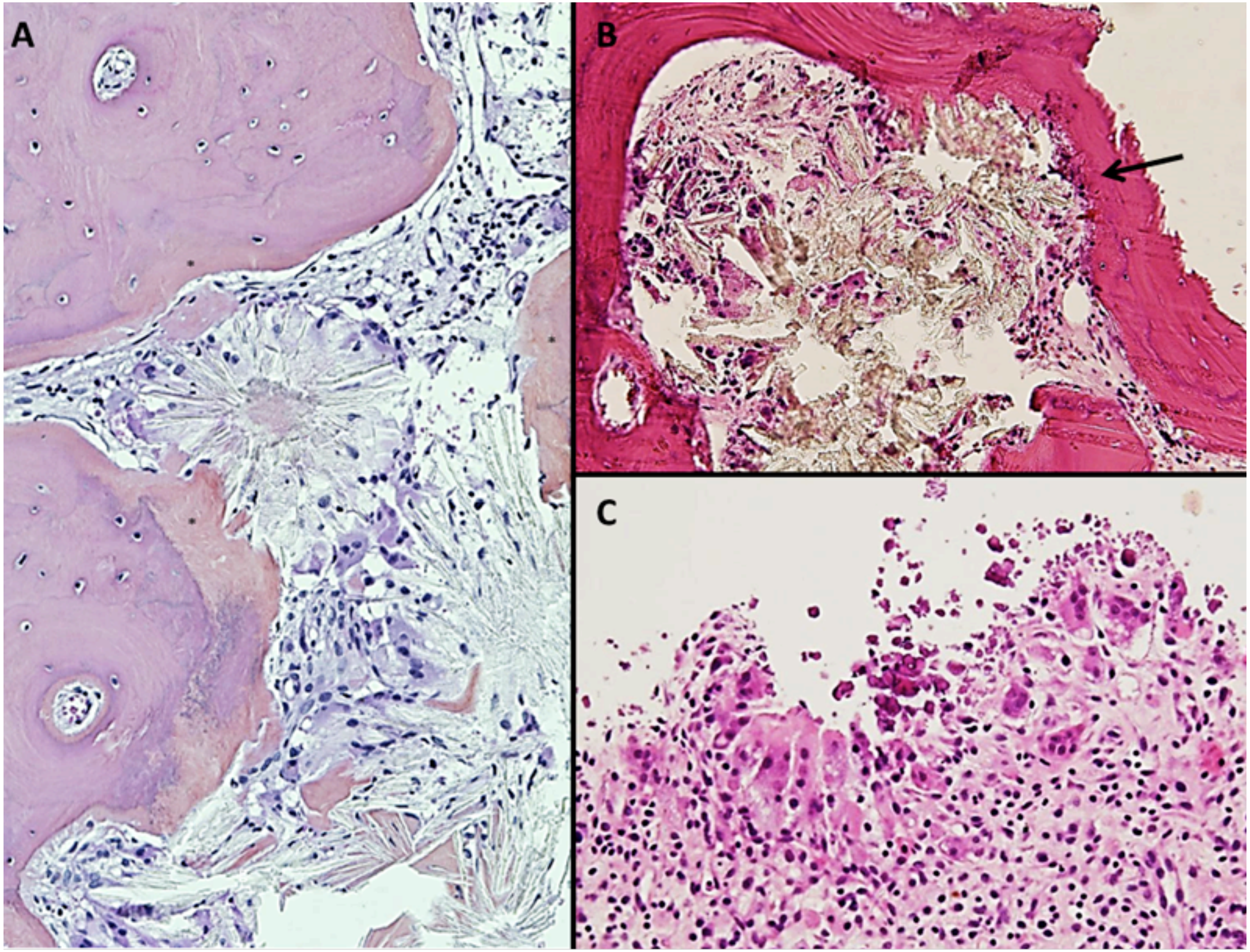

Figure S3

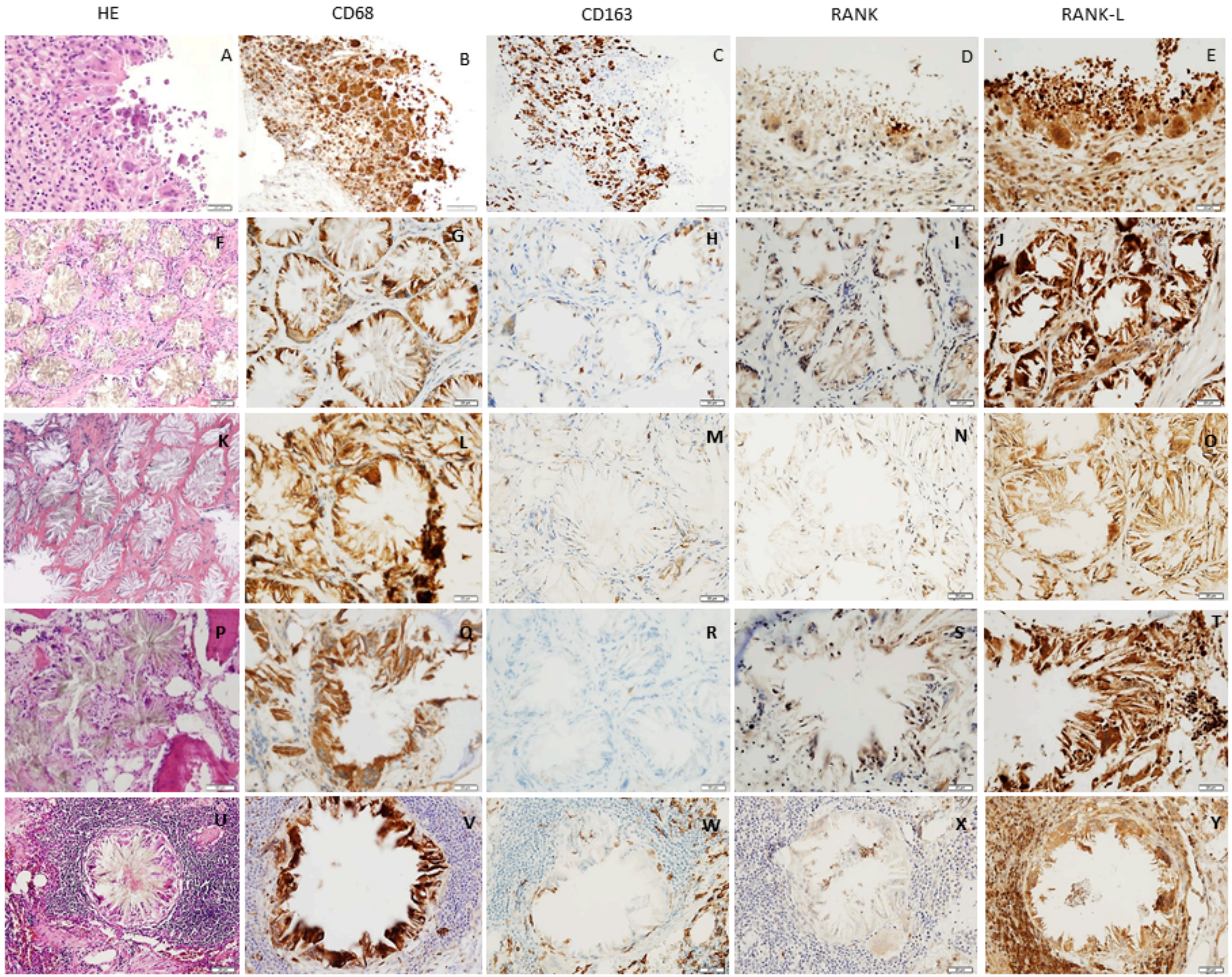

Table S1. Laboratory findings in the five study patients

Longitudinal data are shown with respect to 1) the date liver-kidney transplantation or liver transplantation (D0) and 2) the date of FDG-PET/CT ([F])

	Patient #1							Patient #2					Patient #3				Patient #4				Patient #5			
Date (year)	2013 (Y-3)	2016 D0 LKT	2016 M2	2017 M8	2017 M12[F]	2018 M23[F]	2020 M54[F]	2017 (M-4)[F]	2018 D0 LKT	2018 M6	2019 M12	2020 M24[F]	2015 (Y-3)	2018 D0 LKT	2019 M14[F]	2020 M24	2016 M-10	2017 D0 LT	2017 M6	2019 M36[F]	2017 Y-2	2019 M3	2020 M9[F]	2020 M16
S calcium, mmol/L	2.78	2.61	2.16	2.55	3.38	2.63	2.56	2.46	2.50	2.51	2.38	2.98	2.84	3.18	NA	2.52	1.88	1.93	2.38	2.07	2.45	2.54	2.28	2.35
S corrected calcium, mmol/L	3.06	3.06	2.54	2.90	3.61	2.91		2.79	2.78	2.89	2.60	3.51	3.11	3.53	NA	2.62	2.28	2.44	2.88	2.73	2.57	2.74	2.64	2.59
S ionized calcium, mmol/L	NA	1.40	NA	1.48	1.64	1.50	1.33	1.32	1.34		1.29	1.56		1.39	1.46	1.39	-	-		-				1.24
S albumin, g/L	29	22	25	26	31	29	30	27	29	25	26	19	29	26	28	36	24	19.5	20.2	13.8			28	27
S-Creatinine, µmol/L	694	460	138	162	214	201	271	534	-	510	-	-	-	-	150	184	-	HD	HD	HD			304	199
GFR (CKD-EPI)	HD	HD	33	28	20	21	14	HD	HD	HD	HD	HD	HD	HD	49	38	HD	-	-	-				
C-reactive protein, mg/L	<4	5.6	<4	21	<4	29	6	105	76	14	64	89		27	5	20	31.2	17	6.8	3.3	2.2	10.2		1.2
ACE, IU/L[1]	NA	NA		80	97	143	118	46		99	51	92		48	66	NA	-	-	-	148				118
1,25 OH_2-vitD, ng/L[2]	56	NA	24	80	29	36	31	128		100	173	70	41		52	112	-	-	-	26				5
25 OH-D, µg/L	22	NA	15	26	84	13	24	24		25	59	11.8	24	13	3	NA	42	41	28	31				28
PTH intact, ng/L	65	NA	127	22	10		124	36	12	64	40	39	96	6	3	NA	425	313	10	181				115
PTH-related peptide					<ref					<ref					<ref									
BAP, µg/L[3]	14.6	NA	102	NA	43	56	63.8	15.8		76	40	15			85	NA	294	115	72	112				
CTX, µg/L (ref <0.7 µg/L)	NA	NA	3.27	NA	0.96	0.75	3.03	1.79		4.3	10.9	0.9			0.8	0.8	-		-					
Osteocalcin, µg/L[4]											258		400[i]											
TRAP5b, IU/L (N <4 IU/L)											161													>30
IL-6, ng/L[5]		12,5				<ref					9.4				NA					35.6				
IL-1 beta, ng/L		NA				<ref					<ref				NA					<ref				
TNF alpha, ng/L		NA				<ref					<ref				NA					<ref				
Plasma oxalate, µmol/L (Normal values <33 µmol/L, i.e., 3 mg/L)	87	10	NA	20	NA	30	48.8	110		26	51	58	16		12	12			9.5	19.5				NA

Abbreviations: S, serum; GFR, glomerular filtration rate; ACE, angiotensin-converting enzyme; PTH, parathyroid hormone; BAP, bone alkaline phosphatise; CTX, C-telopeptide; IL, interleukin; TNF, tumor necrosis factor; LKT, liver kidney transplantation; D, days; M, months; Y, year; NA, not available;.

[1] ACE, angiotensin-converting enzyme; reference range (ref) for cases 1 and 2: 16-85 IU/L, for case 3: 12-68 IU/L, for case 4: 20-70 IU/L.

[2] Ref for 1,25OH_2-vitaminD in the general population according to the assay's manufacturer for cases 1 and 2: 18-60 ng/L; thereafter, 15-90 ng/L (as of 2017) for case 3: 15-90 ng/L, for case 4: 20-80 ng/L. The reference values of 1,25 OH_2-vitaminD while on dialysis are lower than those for the general population

[3] BAP, bone alkaline phosphatase, ref according to the assay: for cases 1 and 2: <14.5 µg/L; thereafter, <27 µg/L (as of 2017), for case 3 <45 µg/L, for case 4: <23 µg/L

[4] Ref for osteocalcin varies according to age from 14 to 42 µg/L, median value in dialysis patients: 200 µg/L (Fusaro, *et al. J Nephrol* 32, 635–643; 2019)

[5] Ref for IL-6 according the to the assay's manufacturer for cases 1 and 2: <3 ng/L, for case 4: < 7 ng/L

Table S2. Summary of imaging (FDG-PET/CT and bone scintigraphy) and histology findings in five patients with severe oxalosis

	Patient #1	Patient #2	Patient #3	Patient #4	Patient #5
**FDG-PET/CT findings**					
Timing of onset in relation to LT/SLKT, months	12 months after SLKT	4 months before SLKT	15 months after SLKT	10 months before LT	One month after SLKT
Presence of diffuse hypermetabolic lesions<br>Localization	Yes<br>- Diffuse skeletal lesions<br>- Spinal lesions (posterior elements)<br>- Numerous joint and muscle insertion lesions<br>- Cutaneous areas (abdomen)	Yes<br>- Diffuse skeletal lesions<br>- Spinal lesions (spondylitis)<br>- Numerous joint lesions (sacroiliitis)<br>- Calcified masses located in the gluteal muscles<br>- Larynx<br>- Native left kidney	Yes<br>- Diffuse skeletal lesions<br>- Spinal lesions (spondylitis)<br>- Numerous joint lesions (sacroiliitis)<br>- Muscle insertion lesions<br>- Cutaneous areas (abdomen)<br>- Larynx	Yes<br>- Diffuse skeletal lesions<br>- Spinal lesions (spondylitis)<br>- Numerous joint lesions (sacroiliitis)<br>- Larynx	Yes<br>- Diffuse skeletal lesions<br>- Spinal lesions<br>- Numerous joint lesions (shoulder girdle, chondrocostal, and costovertebral joints)<br>- Native right kidney
**Last FDG-PET/CT scan**<br>Time after SLKT/LT, months<br>Clinical scenario	53 months<br>Moderately reduced pain, improved mobility.<br>Partially reduced intensity of hypermetabolic bone lesions (sacral bone: decrease of maximum SUV from 10 to 6.7).	24 months<br>Persistent and disabling pain.<br>Mildly reduced intensity of hypermetabolic lesions, reduction in volume of calcified muscle masses.	NA	10 months<br>Partially reduced intensity of hypermetabolic bone lesions (sacroiliitis: decrease of maximum SUV from 11.9 to 5.3).	8 months<br>Lesions with stable tracer avidity.
Cardiovascular calcifications on CT, localization	Iliac artery and aortic valve calcification	Diffuse aortic, iliac, and arteries calcifications; cardiac valve calcifications	Aorta	Iliac artery	None
**Bone scintigraphy**	Mild bone tracer fixation adjacent to calcium deposits	NA	NA	Mild but diffuse tracer uptake in the bone adjacent to calcium deposits	NA
**Histology**	Presence of granulomas surrounding Ca-Ox in the following tissue samples:<br>- Iliac nodes removed during transplantation surgery<br>- Vertabra (L3) showing an increase tracer uptake on FGD-PET after SKLT<br>- Iliac bone biopsy<br>- Hallux exostosis<br>- Granulomas in the kidney graft	- Transiliac bone biopsy performed before SKLT:<br>Ca-ox crystals surrounded by granulomas and an osteoid-like matrix<br>- Biopsy of calcified muscular masses (M12):<br>Calcium deposits surrounded by granulomas; calcium deposits were not organized in crystals ((i.e., absence of birefringence under polarized light microscopy and von Kossa positivity)<br>- Liquid from tophi on the fingers:<br>Calcium-phosphate carbapatite in the absence of Ca-Ox crystals	NA	- Iliac bone biopsy:<br>Ca-Ox crystals surrounded by granulomas and an osteoid-like matrix	- Cutaneous biopsy<br>Ca-Ox crystals surrounded by granulomas<br>- Kidney grafts: rare crystals associated with reactive granulomatosis

Abbreviations: F, female; M, male; LT, liver transplantation; SLKT, simultaneous liver-kidney transplantation; NA, not available; Ca-Ox, calcium-oxalate; SUV, standardized uptake value.

Supplement material: discussion

This is the first study to provide a detailed characterization of the inflammatory reactions occurring during the course of severe SO caused by PH. Systemic tissue Ca-Ox deposition in SO elicited a diffuse and persistent inflammatory response – which was assessed for the first time using FDG-PET/CT imaging. As expected, a prominent involvement of the skeleton was observed in our study; however, this is, to our knowledge, the first research to show that reactive granulomatous inflammation to crystal deposition is likely the key driver for rheumatic pain, fractures, and associated morbidity.

Inflammatory joint involvement during the course of SO is similar to that occurring in other forms of crystal arthropathy (e.g., gout).[S1] Apart from bone and joints, hypermetabolic lesions on FDG-PET/CT imaging were identified in numerous other tissues– including muscles, cartilages, cutaneous areas, and the kidneys. These imaging findings confirm previous experimental data supporting a key role for inflammation in the pathogenesis of renal injury during the course of both renal oxalosis[4] and other crystal nephropathies.[S2]

Herein, FDG-PET/CT findings – as well as the results of histology and laboratory examinations – consistently demonstrated that bone granulomas promoted the erosion of the adjacent osseous tissue. These alterations, which were reflected by the persistently elevated serum levels of bone turnover biomarkers, resulted in a high occurrence of fractures. In accordance with previous bone biopsy studies,[2] our histology results showed that granulomas induced by Ca-Ox deposition consisted mainly of macrophages and MGC that exhibited osteoclast-like activity. Both cell types expressed high levels of CD68 and RANK-L. The ability of RANK-L and its cell surface receptor RANK in inducing an osteoclast phenotype in monocytes/macrophages is widely recognized.[S3] Collectively, our current findings support a key role for this signaling pathway in mediating the increased bone resorption and the high risk of fractures occurring in SO.

The presence of a granulomatous inflammatory response in this clinical entity was further confirmed by elevated serum levels of specific biochemical markers (i.e., ACE and/or $1,25OH_2D$) – which may be consequent to ACE and 1-alpha hydroxylase hyperexpression in cells of macrophagic lineage. Another laboratory hallmark observed in our patients was hypoalbuminemia. The preservation of nutritional status at the beginning of follow-up raises the possibility that hypoalbuminemia may be driven by inflammation. Interestingly, a subset of patients had laboratory evidence of a chronic state of inflammation as reflected by elevated serum CRP and/or IL-6 levels. Our study also provides biological plausibility for an association between the amount of Ca-Ox deposition as well as the time on dialysis and the extent of granulomatous inflammation.

Patients with severe SO also exhibited hypercalcemia – which can nonetheless be underdiagnosed based on serum total calcium (owing to the presence of hypoalbuminemia). Hypercalcemia has been previously reported in certain cases of systemic oxalosis.[5] This condition typically appears after several years on dialysis and is generally paralleled by the onset of rheumatic pain. In line with previous observations, [5] hypercalcemia was PTH-independent in our study patients. Hypercalcemia is involved in the pathogenesis of valvular and vascular calcifications and may also promote the urinary crystallization of calcium oxalate. The concomitant presence of different alterations can explain the occurrence of hypercalcemia in SO. First, granuloma-induced bone resorption may lead to the release of $CaCO_3$ in the bloodstream. Second, increased $1,25OH_2D$ serum levels can promote calcium intestinal absorption. Finally, calcium ions may leak from the dissolution of systemic Ca-Ox deposits. MGC may play a role in the dissolution of systemic Ca-Ox crystals – which may in turn lead to high urinary and plasma levels of oxalate and calcium.

The study patients presented severe oxalosis due to a prolonged time on dialysis; consequently, the imaging and laboratory hallmarks described in this study may be absent in patients with primary hyperoxaluria with no or mild oxalosis. Despite the small sample size, our study may have significant implications for the clinical management of patients with SO. First, our data support the utility of FDG-PET/CT imaging,

inflammatory biological markers, and hypercalcemia to assess the presence and the severity of the systemic reactive inflammation. FDG-PET/CT before transplantation may help clinicians to assess the severity of oxalosis in primary hyperoxaluria (both type 1 and type 2) and the related risk of recurrent crystal nephropathy in the post-transplantation period. In general, primary hyperoxaluria type 2 is characterized by a less severe clinical course compared with type 1. However, there have been reports of patients with primary hyperoxaluria type 2 who required kidney transplantation[6] and, for that reason, larger prospective cohorts are needed. Second, the biochemical quantification of serum ionized calcium and bone turnover markers may improve the capacity to diagnose hypercalcemia and assess the risk of fracture. Third, our results may have therapeutic implications. We found that bone antiresorptive agents can be part of the therapeutic armamentarium to control hypercalcemia. High steroid doses were also effective to control hypercalcemia – a finding consistent with other case reports.[5] However, hypercalcemia invariably relapsed after drug withdrawal – ultimately requiring prolonged treatment. With regard to crystal rheumatism, high-dose corticosteroids may be temporary effective as it was observed in patient #2. Drugs targeting inflammation (e.g., anti-IL-1 and IL-6 agents) may hold promise to treat calcium crystal arthropathy.[7] Unfortunately, the risk of infectious complications is not negligible and, for that reason, additional studies are necessary.

SUPPLEMENTARY REFERENCES

S1. McCarthy GM, Dunne A. Calcium crystal deposition diseases—beyond gout. Nat Rev Rheumatol. 2018;14:592–602. https://doi.org/10.1038/s41584-018-0078-5.

S2. Mulay SR, Anders HJ. Crystal nephropathies: mechanisms of crystal-induced kidney injury. Nat Rev Nephrol. 2017;13:226–240. https://doi.org/10.1038/nrneph.2017.10.

S3. Ono T, Hayashi M, Sasaki F, Nakashima T. RANKL biology: bone metabolism, the immune system, and beyond. Inflamm Regen. 2020;40:2. https://doi.org/10.1186/s41232-019-0111-3.
